# Non-reproductive triggers of postpartum psychosis

**DOI:** 10.1007/s00737-016-0674-9

**Published:** 2016-10-08

**Authors:** Ian Brockington

**Affiliations:** University of Birmingham, Edgbaston, Birmingham, HR7 4TE UK

**Keywords:** Bromocriptine, Post-operative psychosis, Corticosteroid therapy, Thyrotoxicosis

## Abstract

Bipolar disorders, and other psychoses, are known to be triggered by a number of agents apart from the reproductive process. In some women, pregnant or recently delivered, psychosis may be due to these alternative triggers. There are substantial numbers of mothers suffering from childbearing psychoses, who have been prescribed bromocriptine or steroids, have had surgical operations or developed thyrotoxicosis. It is best to eliminate these episodes and cases from study samples of puerperal psychosis.

## Introduction

Many perhaps think of ‘puerperal psychosis’ as triggered by the cascade of hormones after childbirth. In *What is Worth Knowing about* ‘*Puerperal Psychosis*’ (Brockington 2014), it is proposed that the early postpartum trigger is only one of a group of triggers acting at various times in the reproductive process, including pregnancy, later in the postpartum period, after weaning, after abortion and at a stage in the menstrual cycle.

But in addition to all these triggers acting on the bipolar/cycloid diathesis, there are several non-reproductive triggers, which may result in psychoses that present in pregnancy or the puerperium.

## Bromocriptine

The argoline derivative, 2-bromo-α-ergocriptine, a dopamine D_2_ agonist, was developed in 1968 to block the release of prolactin (Parkes 1979), and has been used since 1978 in a dose of 5–7.5 mg/day to inhibit puerperal lactation. Since then, 14 postpartum episodes have been reported, associated with its use (Brook and Cookson 1978; Vlissides et al. 1978; Charbonnier and Planche 1981; Canterbury et al. 1987; Kemperman and Zwanikken 1987; Daw 1988; Iffy et al. 1989; Durst et al. 1990; Fisher et al. 1991; Reeves and Pinkofsky 1997; Pinardo Zabala et al. 2003; Misdrahi et al. 2006). In a fifteenth (Lake et al. 1987), the mother became overactive before bromocriptine was prescribed. The onset, where known, was early in the puerperium. In eight, the clinical picture was typical of mania, and another ran a bipolar course, starting with depression (Reeves and Pinkofsky 1997). In three, it was atypical (Daw 1988; Pinardo Zabala et al. 2003), including one with a seizure (Iffy et al. 1989), and, in one, depressive, with ‘voices’ telling her to destroy the baby (Canterbury et al. 1987). In six, duration was short—1 week or less in five, and 17 days in the sixth. Two mothers recovered after withdrawal of bromocriptine without other treatment (Charbonnier and Planche 1981; Canterbury et al. 1987); but other episodes have lasted up to 2 months, and required much treatment, in one case ECT. Two mothers suffered previous manic episodes, including steroid-triggered episodes (Fisher et al. 1991; Misdrahi et al. 2006). One had a family history of psychosis (Fisher et al. 1991).

In my series, there was one possible example:

A 24-year old was delivered of her 1^st^ child by Caesarean section because of a suspected foetal facial cyst. She was treated with bromocriptine 5 mg/day. She felt excited because the baby was normal. On day 4 she began to behave strangely and became increasingly anxious, overactive, emotionally labile and disinhibited. She was talkative and cheerful, and gave a running commentary on her actions. Admitted to hospital, she was confused and perplexed; she did not know the day of the week. She was unable to think clearly, and spoke little, with slow and deliberate answers. The possibility of a toxic delirium secondary to bromocriptine was raised; it was stopped and she was treated with chlorpromazine. She rapidly recovered, but, a month later, was ‘over the top’ for two days. She remained well in the next 32 years.

It is clear that bromocriptine can trigger postpartum episodes, perhaps only in mothers with a bipolar diathesis. It can also trigger episodes in other circumstances, as described in the treatment of parkinsonism (Lipper. 1976), acromegaly (Le Feuvre et al. 1982; Valdes et al. 1989) and prolactinoma (Turner et al. 1984). Psychiatric complications required withdrawal of bromocriptine in 5/66 cases (Serby et al. 1978).

## Post-operative psychosis

There is a scanty literature on the occurrence of psychosis after surgical operations (Abdullah et al. 2006). Because of the great variety of surgical procedures, the range of disorders that require them and the complications of anaesthetics and analgesics, this is a complex subject; for example, there is a literature on the psychoses that follow surgery for temporal lobe epilepsy, and there are post-operative confusional states. In the literature on childbearing psychoses, there are only eight instances of the association of postpartum and any form of post-operative psychosis: three followed sterilization (Ellery 1927; Hess 1938; Van Steenbergen 1941), two occurred after oophorectomy (Robertson Blackmore et al. 2008) and three after other surgical operations (Heidema 1932; Hess 1938; Van Steenbergen 1941). It is impossible to evaluate these because there is no information on the number of operations performed. This information, however, was available in my series—46 surgical operations were known to have been performed during the study period. Five mothers had post-operative episodes, including this mother who had only three psychotic episodes in 39 years, one after one of her three births and two after surgical operations:

A 25-year old developed a puerperal cycloid episode, with onset day 9, after her 1^st^ birth. She had only two other episodes, both following surgery: after hysterectomy she was briefly hypomanic, and after a double knee replacement she suffered an acute psychosis, with pressure of speech, incoherence, violence and delusions of reference, poisoning and jealousy.

The frequency 5/46 (11 %) is about the same as post-abortion episodes (14 %).

It should be noted that many mothers were delivered by Caesarean section or forceps under general anaesthesia; in my bipolar/cycloid group, this was the mode of delivery in 30 episodes. Postpartum psychoses after Caesarean section could be post-operative rather than puerperal psychoses.

## Corticosteroid therapy

This was introduced into therapeutics in 1950. There is a scattered literature on the precipitation of mania, severe depression, paranoid disorders, delirium and other psychoses by adrenal corticosteroids (for example, Sirois 2003; Kenna et al. 2011); about half of the reported cases are bipolar. One would, therefore, expect an association with postpartum bipolar disorders. In the literature, there are two cases of this association (Svoboda 1957; Johnson 1996), to which I can add six, which are briefly summarized below:

A 29-year old became pregnant for the 1^st^ time. Because of foetal distress, she was delivered @ 35 weeks gestation by emergency Caesarean section. She had two doses of dexamethasone at the time of delivery. On day 2 she became sleepless and began writing copious notes. By day 7 she became overactive and aggressive, and said the television and radio referred to her, and her husband was trying to infect her and the baby with AIDS, which he had contracted during an affair. She later had a second episode of postpartum mania, with onset six weeks after the birth.

A 35-year old, after six years of infertility, conceived with gamete intra-fallopian transfer, and became pregnant with twins. At 30 weeks gestation, she was given dexamethasone to increase the maturity of the foetal lungs. A week later she was admitted to the maternity hospital with antepartum bleeding. She became excited, elated and sleepless and expressed persecutory ideas. She was talking constantly, giving a running commentary on everything that was happening. At 32 weeks gestation, she gave birth; one of the twins had Down’s syndrome. Three weeks later her psychosis recurred, with head-banging, restlessness, insomnia and racing thoughts. On admission to hospital she was depressed, and did not recover until five months later. She remained well for the next 20 years.

A 28-year old gave birth to her 1^st^ child. For two weeks she was ‘on a high’, talking non-stop. Six weeks later she developed pityriasis rosea, treated with steroids. She became very high, ‘brilliant’, her mind racing. This lasted a week until she stopped the steroids. She then became depressed for a year. Her baby also became high on steroids.

A 35-year old, who for several years had suffered from poly-arthritis and Crohn’s disease, developed pre-eclamptic toxaemia during her 1^st^ pregnancy, and was delivered by Caesarean section. On day 12 she developed a cycloid psychosis – slow, confused and perplexed – from which she recovered within three weeks. When the baby was four months old, her arthritis recurred. Treated with ibuprofen, she developed a purpuric vasculitis with bullous lesions (Stevens-Johnson syndrome), together with laboratory evidence of systemic lupus erythematosus. She was treated with prednisolone 60 mg/day. Within four days she became withdrawn and mute, staring into the distance. She washed obsessively, complaining of sweating and halitosis. A CAT scan showed diffuse cerebral abnormalities, and an EEG low frequency activity. She was again treated with steroids, and recovered in three months.

A 24-year old, whose mother suffered from puerperal psychosis after her own birth, became pregnant for the 1^st^ time. Two days before the onset of labour she developed a rash, which was treated with prednisone 20 mg/day. After the birth, on day 7, she became weepy, and disorientated, then elated and confused. She felt her brain was exploding. She thought her partner was trying to kill her and believed she was the Messiah. She telephoned a minister to say she had the solution to the Irish problem. She was writing reams of gibberish, which she believed was important to the future of humanity. After recovery she suffered severe bonding problems, which continued after the second baby was born. In 28 years she suffered one further manic episode.

A 28-year old, @ 35 weeks gestation, was treated with prednisolone for idiopathic thrombocytopenia. At 39 weeks she was delivered by forceps, and steroids were discontinued. On day 3 she became agitated, weepy and perplexed, with confusion and ‘paranoid’ ideation. On admission to hospital, prednisolone was started again. She remained retarded, vague, staring into space, suspicious, speaking in a monotonous voice of being evil, hopeless and a failure. She denied the existence of her husband and the baby. She suddenly disrobed and started shouting, “Let me die!” Seven days later she abruptly improved. Two weeks later steroids were stopped, but soon started again because a diagnosis of systemic lupus erythematosus was made. She had no further episode in the course of nine years.

Only four other mothers were treated with steroids in any form. Furthermore, these six mothers, in a total of 88 years observation, had, apart from those associated with childbearing or steroid therapy, only one psychotic episode between them. It seems best to regard these episodes as steroid psychoses and not childbearing psychoses.

## Thyrotoxicosis

Note that this patient had myxoedema disease is occasionally associated with acute psychosis, as shown by at least nine case reports and Brownlie’s survey in New Zealand (Brownlie et al. 2000): three patients have recovered after surgical treatment (Bursten 1961; Lazarus and Jaffe 1986; Abbasi et al. 2009), and others after treatment with propylthiouracil (Øestergaard Jensen 1950) or propranolol (Lee et al. 1991). Although occasional cases had manic features (Bursten 1961; Stowell and Barnhill 2005), a wide variety of other psychotic syndromes have been seen.

Of particular interest are two episodes of thyroid disease that developed concurrently with late onset postpartum psychoses (Bokhari et al. 1998; Stowell and Barnhill 2005):

Eleven weeks after her 3^rd^ child was born, a 29-year old developed insomnia, weight loss and fatigue intolerance. She appeared confused and was disorientated in time and place. She heard Jesus talking to her, and also had visual hallucinations. She believed she was pregnant with the Christ child, and would be killed by hospital staff. She had thyrotoxicosis, associated with thyroiditis. Her psychiatric symptoms improved concurrently with its treatment.

A 35-year old gave birth to twins. Seven months later she presented with increased energy, lack of the need for sleep, racing thoughts, preoccupation with bible reading, and the belief that God had fathered her babies. She collapsed and was admitted. She had myxoedema due to postpartum thyroiditis. With thyroxin and risperidone she recovered within six days.

Table 1 shows the details of eight other mothers with some evidence of this association.

**Table 1:** Association with thyrotoxicosis

First author	Clinical features	Evidence of thyrotoxicosis	Comment
Johnstone 1884	Onset of psychosis 6 months after the birth of her third child	Signs of exophthalmic goitre developed concurrently	Late postpartum onset
Knauer 1897 Case 65	Onset of a chronic depressive psychosis after her sixth birth	She developed thyrotoxicosis	No data on timing
Sivadon 1933 Case 15	Onset of psychosis on day 9 after her first birth	She had an enlarged thyroid and tremor. She also had a fever of 38°	She died on day 22, infection possible
Schröder 1936 case 38	She gave birth at 38, and on day 11 developed a psychosis	She had a goitre at the age of 10 and puerperal psychosis at 38; 7 months later, while still psychotic, she was noted to have slight exophthalmos and sweaty hands	Thyrotoxicosis was noticed 7 months later
Abély et al. 1947	Onset of psychosis shortly after the second, third, fourth and fifth births	During two episodes, thyroid enlargement was noted. During the fourth episode, tests showed transitory hyperthyroidism, whose disappearance coincided with improvement in the clinical state	Only the fourth episode of postpartum psychosis was affected
Retzeanu et al. 1960	Onset of psychosis in the ninth month of gestation	She had a large soft thyroid and tachycardia; she refused surgery on her goitre and was treated with psychotropic drugs and radioactive iodine. Improvement in mental state and reduction in goiter were concurrent	Prepartum psychosis, with a possible response to anti-thyroid treatment. She also had pleurisy and galactorrhoea
Butts 1968	Psychosis of unknown onset after first birth, and with onset day 7 after the second	During the second episode, she had an enlarged thyroid, systolic murmur and tremor; but her protein-bound iodine was only 4.4 μg/100 ml and her basal metabolic rate minus 12 %	Only the second episode of postpartum, psychosis was affected. Evidence of thyrotoxicosis was equivocal
From my series	Onset of depressive psychosis 7 months after the birth	Goitre, loss of weight and tremor during her second pregnancy	Late postpartum onset, response to ECT
	Psychosis on day 5 after both her first and second births	She had a goitre and clinical signs; the diagnosis was confirmed by laboratory tests. Treatment included radioactive iodine	Only the second episode of postpartum, psychosis was affected

In all, there are 11 mothers with concurrent childbearing psychosis and thyrotoxicosis; 1 had prepartum, 4 late postpartum, 5 early postpartum and 1 unknown onset. Three had other episodes of postpartum psychosis that were not accompanied by thyrotoxicosis. One mother may have responded to thyroid treatment alone. There is no consistency in these data. It is possible that thyrotoxicosis is a factor acting synergistically with childbearing factors, but more evidence is required.

## Discussion

Unless Caesarean section is included among the surgical procedures, there are not many ‘non-reproductive’ triggers of childbearing psychoses, but any steps taken to improve the homogeneity of samples will be beneficial (Brockington 2016). Where these triggers are associated with episodes, they should be discounted, and where these are the sole episodes in the mother’s history, her case should be removed.

## References

[CR1] Abbasi B, Sharif Z, Sprabery L (2009). Hypokalemic thyrotoxic periodic paralysis with thyrotoxic psychosis and hypercapnic respiratory failure. Am J Med Sc.

[CR2] Abély P, Sizaret P, Laine B (1947) Considérations sur un état maniaque post-puerpéral récidivant. Ann Méd Psychol 105:379–382

[CR3] Abdullah MS, Al-Waili NS, Baban NK, Butler GL, Sultan L (2006). Postsurgical psychosis: case report and review of literature. Adv Ther.

[CR4] Bokhari R, Bhatara VS, Bandettini F, McMillin JM (1998). Postpartum psychosis and postpartum thyroiditis. Psychoneuroendocrinology.

[CR5] Brockington IF (2014). What is Worth Knowing about ‘Puerperal Psychosis’.

[CR6] Brockington IF (2016) The Psychoses of Menstruation and Childbearing. Cambridge University Press, Cambridge

[CR7] Brook NM, Cookson IB (1978). Bromocriptine-induced mania?. Br Med J.

[CR8] Brownlie BE, Rae AM, Walshe JW, Wells JE (2000). Psychoses associated with thyrotoxicosis—‘thyrotoxic psychosis’: a report of 18 cases with statistical analysis of incidence. Eur J Endocrinol.

[CR9] Bursten B (1961). Psychoses associated with thyrotoxicosis. Arch Gen Psychiatry.

[CR10] Butts NF (1968). Psychodynamic and endocrine factors in postpartum psychoses. J Natl Med Assoc.

[CR11] Canterbury RJ, Haskins B, Kahn N, Saathoff G, Yazel JJ (1987). Postpartum psychosis induced by bromocriptine. South Med J.

[CR12] Charbonnier JF, Planche R (1981). Bromocriptine et manie du post-partum. Actualités Psychiatriques.

[CR13] Daw JL (1988). Postpartum depression. South Med J.

[CR14] Durst R, Dorevitch A, Ghinea C, Ginath Y (1990). Bromocriptine-associated postpartum psychotic exacerbation. Harefuah.

[CR15] Ellery RS (1927). Psychosis of the puerperium. Med J Aust.

[CR16] Fisher G, Pelonero AL, Ferguson C (1991). Mania precipitated by prednisone and bromocriptine. Gen Hosp Psychiatry.

[CR17] Heidema ST (1932). Puerperaalpsychosen. Psychiatrische en Neurologische Bladen.

[CR18] Hess M (1938). Über die sogenannten Puerperalpsychosen.

[CR19] Iffy L, Lindenthal J, Szodi Z, Griffin W (1989). Puerperal psychosis following ablaction with bromocriptine. Med Law.

[CR20] Johnson I (1996). Steroid-induced prepartum psychosis. Br J Psychiatry.

[CR21] Johnstone JC (1884). Case of exophthalmic goiter with mania. J Ment Sci.

[CR22] Kemperman CJF, Zwanikken GJ (1987). Psychiatric side effects of bromocriptine therapy for postpartum galactorrhoea. J R Soc Med.

[CR23] Kenna HA, Poon AW, de los Angeles CP, Koran LM (2011). Psychiatric complications of treatment with corticosteroids: review with case report. Psychiatry Clin Neurosci.

[CR24] Knauer O (1897) Über Puerperale Psychosen für practische Ärzte. Karger, Berlin

[CR25] Lake CR, Reid A, Martin C, Chernow B (1987). Cyclothymic disorder and bromocriptine: predisposing factors for postpartum mania. Can J Psychiatry.

[CR26] Lazarus A, Jaffe R (1986). Resolution of thyroid-induced schizophreniform disorder following subtotal thyroidectomy: case report. Gen Hosp Psychiatry.

[CR27] Lee S, Chow CC, Wing JK, Leung CM, Chiu H, Chen CN (1991). Mania secondary to thyrotoxicosis. Br J Psychiatry.

[CR28] Le Feuvre CM, Isaacs AJ, Frank OS (1982). Bromocriptine-induced psychosis in acromegaly. Br Med J.

[CR29] Lipper S (1976). Psychosis in patient on bromocriptine and levodopa with carbidopa. Lancet.

[CR30] Misdrahi D, Chalard R, Verdoux H (2006). Épisode maniaque induit par la bromocriptine en post-partum: à propos d’un cas. Journal de Gynécologie, Obstétrique et Biologie Réproductive.

[CR31] Øestergaard Jensen O (1950). Thyreotoxisk krise simulerende acut psykose: to tilfaelde. Ugeskr Laeger.

[CR32] Parkes D (1979). Bromocriptine. N Engl J Med.

[CR33] Pinardo Zabala A, Alberca Munoz ML, Gimenez Garcia JM (2003). Psicosis posparto asociada a bromocriptina. Anales Medica Interna.

[CR34] Reeves RR, Pinkofsky HB (1997). Postpartum psychosis induced by bromocriptine and pseudoephedrine. J Fam Pract.

[CR35] Retzeanu A, Tomorug E, Elias S (1960). Psihoza periodică la o hipertiroidină în timpul sarcinii. Neurologia Psychiatria Neurochirurgia.

[CR36] Robertson Blackmore E, Craddock N, Walters J, Jones I (2008). Is the perimenopause a time of increased risk of occurrence in women with a history of bipolar affective postpartum psychosis? A case series. Arch Womens Ment Health.

[CR37] Schröder P (1936). Über Wochenbettpsychosen und unsere heutige Diagnostik. Allgemeine Zeitschrift für Psychiatrie.

[CR38] Serby M, Angrist B, Lieberman A (1978). Mental disturbances during bromocriptine and lergotrile treatment of Parkinson’s. Am J Psychiatry.

[CR39] Sirois F (2003). Steroid psychosis: a review. Gen Hosp Psychiatry.

[CR40] Sivadon P (1933). Les psychoses puerperales et leurs séquelles.

[CR41] Stowell CP, Barnhill JW (2005). Acute mania in the setting of severe hypothyroidism. Psychosomatics.

[CR42] Svoboda L (1957). Laktační psychosa při lupus erythematodes léčeném ACTH. Cesk Psychiatr.

[CR43] Turner TH, Cookson JC, Wass JAH (1984). Psychotic reactions during treatment of pituitary tumours with dopamine agonists. Br Med J.

[CR44] Valdes J, Otazo R, Bulnes J (1989). A case of toxic psychosis caused by bromocriptine]. Actas Luso-españolas de Neurologia, Psiquiatria y Ciences Afines.

[CR45] Van Steenbergen-van der Noordaa MC (1941). Generatie-psychosen.

[CR46] Vlissides DN, Gill D, Castelow J (1978). Bromocriptine-induced mania?. Br Med J.

